# Supplementary material for: Associations between Parental Educational Attainment, Children’s 24-h Behaviors and Children’s Hyperactivity Behavior in the COVID-19 Pandemic
Source: Healthcare (Basel). 2024 Feb 21;12(5):516. doi: 10.3390/healthcare12050516 (PMC10930545; doi:10.3390/healthcare12050516)
Supplement: Supplementary file 1 [file healthcare-12-00516-s001.zip › healthcare-2810919-supplementary.pdf]

Supplementary-A: Data filtering

The data cleaning process for the online questionnaire package involved addressing data completion time, duplicated data, and abnormal or improbable data.

As the questionnaire was completed through an online link, the data were reviewed for completion time. The average completion time for the questionnaire was 1247 seconds, with a standard deviation was 482.25. To ensure the validity of the data, completion times within a single sitting were considered reasonable between 360 and 480 seconds. In this study, questionnaire completion times ranged from 360 to 2211.5 seconds.

Duplicate entries were handled by selecting entries that fell within the acceptable completion time range. If duplicate entries had completion times within this range, only the first entry was retained.

Unusual or improbable data were addressed in accordance with the key variables of interest in the study–sleep time, physical activity time, and digital media use time.

For sleep (SL) duration among children aged 6–12 years, the standards of the American Academy of Sleep Medicine (AASM) and the Ministry of Education (MOE) of the People's Republic of China were taken into account [51,52]. The AASM recommends 9–12 hours of sleep, while the MOE recommends a minimum of 10 hours for this age group. To allow for variability, a range of 2 hours within the age-specific sleep recommendation was included for analysis. Consequently, the final SL time range was set at 7 hours $\leq$ SL $\leq$ 12 hours. Physical activity (PA) and digital media (DM) time were determined based on sleep time within a 24-hour day. Given the rarity of PA durations being 0, the PA time range was set at 0 $<$ PA $\leq$ 17 hours. To ensure data accuracy, the DM use time was defined within the range of 0 hours $\leq$ DM $\leq$ 17 hours, without exceeding the mean time (1.12 hours) + 2 standard deviations (0.9 hours). As a result, the final DM use time range was established as 0 hours $\leq$ DM $\leq$ 2.92 hours.

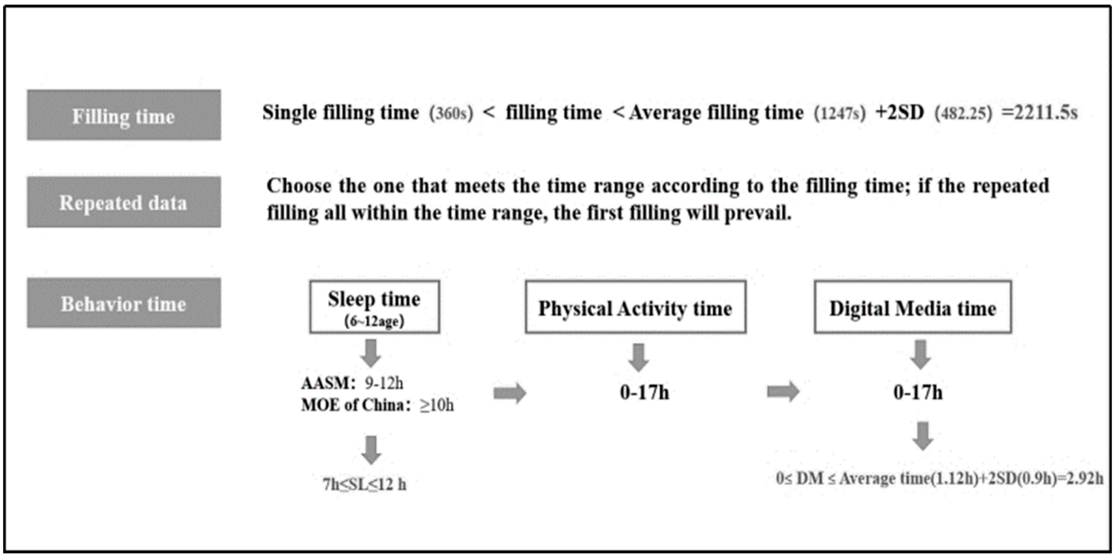

Filling time
Single filling time (360s) < filling time < Average filling time (1247s) +2SD (482.25) =2211.5s
Repeated data
Choose the one that meets the time range according to the filling time; if the repeated filling all within the time range, the first filling will prevail.
Behavior time
Sleep time (6~12age)
AASM: 9-12h
MOE of China: ≥10h
7h≤SL≤12 h
Physical Activity time
0-17h
Digital Media time
0-17h
0≤ DM ≤ Average time(1.12h)+2SD(0.9h)=2.92h

Supplementary-B: Survey instruments

Survey Instructions

1. This survey is to be completed by parents with primary school children **aged 7–12 years.**
2. If you have **more than one child in primary school**, please answer in relation to only **one child (i. e. select only 1 child)**
3. It will take about **20 minutes** to complete.
4. Your answers are very important to us. There are no right or wrong answers. Please answer as best as you are able to.

**Part 1 Survey of digital media habits (Parent-smalQ33®, Parent Surveillance of Digital Media Habits in Childhood Questionnaire®)**	
No.	Question
	**Home Environment**
1.	Which digital media device/s are accessible to your child at home for his/her use? (You can select more than one option)<br>☐ Computer (e.g., desktop, laptop)<br>☐ Mobile devices (e.g., smartphone, tablet)<br>☐ Television (e.g., viewing programs, accessing the Internet, playing games)<br>☐ Video game devices (e.g., Xbox, PlayStation, Nintendo, Wii)<br>☐ Others: _____ (Please specify)<br>☐ None
2.	Which digital media device does your child own (exclusive use or shared with siblings)<br>☐ Computer (e.g., desktop, laptop)

	☐ Mobile devices (e.g., smartphone, tablet)<br>☐ Television (e.g., viewing programs, accessing internet, playing games)<br>☐ Video game devices (e.g., Xbox, PlayStation, Nintendo, Wii)<br>☐ Others: _____ (Please specify)<br>☐ None
	**Parent Digital Media Habits**<br>***For questions 3 and 4, recall a normal day in the last week.*** *(Normal means an ordinary day without special events or occasions)*
3.	How much time do you spend on digital media for these activities on a **WEEKDAY**?<br>*[hr/min]*<br>▪ Entertainment (e.g., watching videos and movies for entertainment)<br>▪ Playing games<br>▪ Reading (e.g., news, blogs, etc.)<br>▪ Shopping online<br>▪ Social media (e.g., Wechat and tiktok etc.)<br>▪ Work-related activities (e.g., Zoom, Tencent conference, nail platform use)<br>▪ Others: _____ (Please specify)
4.	How much time do you spend on digital media for these activities on a **WEEKEND**?<br>*[hr/min]*<br>▪ Entertainment (e.g., watching videos and movies for entertainment)<br>▪ Playing games<br>▪ Reading (e.g., news, blogs, etc.)<br>▪ Shopping online

- Social media (e.g., Wechat and tiktok etc.)
- Work-related activities (e.g., Zoom, Tencent conference, nail platform use)
- Others: _____ (Please specify)

Parent Perception

5. What role does digital media play in your relationship with your child? (You can select more than one option)

- ☐ Communication (e.g., WhatsApp, Line, WeChat)
- ☐ Digital nanny (e.g., preventing boredom/unwanted behavior)
- ☐ Platform for parent-child bonding (e. g., playing games/shopping/watching videos together)
- ☐ Reward/punishment (e.g., rewarding or withholding use for discipline)
- ☐ Co-parenting (e.g., role of teaching values, educating child)
- ☐ Others: __________ (Please specify)

6. How **beneficia**l you feel your child's digital media use is to them?

	Not beneficial	*Slightly beneficial*	*Fairly beneficial*	*Beneficial*	*Very beneficial*
Communication (e.g., WhatsApp, Line, WeChat)					
Entertainment (e.g., social media, playing video games)					
General knowledge					
Self-expression (e.g., creating art/music)					
School-related work (e.g., homework, project work)					
Others: __________ (Please specify)					

7. How **harmful** you feel your child's digital media use is to them.

	Very harmful l	*Harmful*	*Fairly harmful*	*Slightly harmful*	*Not harmful*
Emotional health (e.g., ability to express and manage emotions appropriately; anger, joy)					
Mental health (e.g., cyber bullying, inappropriate content)					
Social health (e.g., lack of face to face interaction with significant others)					
Physical health (e.g., poor sleep, poor eyesight, lack of physical activity and exercise)					
Privacy of the child (e.g., data mining by vendors)					
Safety (e.g., befriending strangers, exposure to scams)					
Others: _______ (Please specify)					

Child Digital Media Habits

8. How much time does your child access digital media for these activities on a school **WEEKDAY** outside of the school?

[hr/min]

- Accessing information (e.g., google places to go, reading blogs)
- Communication (e.g., social media e.g., Facebook, Twitter, Instagram, Snapchat, etc.)
- Content creation (e.g., making videos, music, or art)
- Entertainment/Leisure (e.g., watching videos, playing games, listening to music, shopping online)
- School-related work (e.g., homework or project work)

	▪ Others: ______ (Please specify)
9.	How much time does your child use digital media for these activities on a **WEEKEND** during a school term<br>*[hr/min]*<br>▪ Accessing information (e.g., Google places to go, reading blogs)<br>▪ Communication (e.g., social media—Facebook, Twitter, Instagram, Snapchat, etc.)<br>▪ Content creation (e.g., making videos, music, or art)<br>▪ Entertainment/Leisure (e.g., watching videos, playing games, listening to music, shopping online)<br>▪ School-related work (e.g., homework, project work)<br>▪ Others: ______ (Please specify)
10.	What limits do you currently set for your child for their digital media use on **WEEKDAYS** (If any)?<br>(You can check more than one option)<br>☐ Disable or remove device from the room during sleep<br>☐ Limit time of usage for entertainment related use (Please specify: ___hr ___min)<br>☐ Limit time of usage for school-related online activities (Please specify ___hr__min)<br>☐ Limit to specific content (e.g., parental controls/blocking inappropriate sites)<br>☐ Move away from a screen every 30 minutes<br>☐ None of the above<br>☐ Others: ______ (Please specify)

11. What limits do you currently set for your child for their digital media use on **WEEKENDS** (If any)?

(You can check more than one option)

- ❑ Disable or remove device from the room during sleep
- ❑ Limit time of usage for entertainment related use (Please specify: ___hr ___min)
- ❑ Limit time of usage for school-related online activities (Please specify ___hr__min)
- ❑ Limit to specific content (e.g., parental controls/blocking inappropriate sites)
- ❑ Move away from a screen every 30 minutes
- ❑ None of the above
- ❑ Others: ______ (Please specify)

12. Compared with the time before COVID-19, What's the change in the average time your child spends on the following activities every day (Think about the past 7 days)?

	Significantly increased	Slightly increased	almost	Slightly decreased	Significantly decreased
Screen usage (computer, smartphone, tablet, watching TV)					
Physical activities (dynamic games, walking, fitness or sports competitions)					
Outdoor activities (outdoor games, fitness or sports competitions)					
Total sleep (including daytime nap)					
Quality of life					

	**Child Physical Play Habits**
13.	Think of the last 7 days. How much time did your child spend on ***outdoor*** **play** on a typical school **WEEKDAY**? (Not including school hours)<br>___hr___min
14.	Think of the last 7 days. How much time during ***outdoor*** **play** on a **WEEKDAY** was your child engaging in *moderate to vigorous activity? (activities which make your child breathe heavier and faster)<br>___hr ___min (Please estimate)
15.	Think of the last 7 days. How much time did your child spend on ***outdoor*** **play** on a typical school **WEEKEND?**<br>___hr___min
16.	Think of the last 7 days. How much time during ***outdoor*** **play** on a **WEEKEND** was your child engaging in *moderate to vigorous activity? (Activities which make your child breathe heavier and faster)<br>___hr ___min (Please estimate)

17.	What physical activity guidelines do you follow in relation to your child?<br>☐ Accumulate <u>at least</u> 60 mins of moderate to vigorous activity* everyday<br>☐ Break up sedentary periods lasting longer than 90 minutes with 5-10 minutes of standing and moving around/play<br>☐ Perform 2–3 times a week of resistance exercise (e.g., push ups, sit ups, etc)<br>☐ Others: ______ (Please specify)<br>☐ Not aware of guidelines<br>*Moderate to vigorous activities are those which cause noticeable increases in breathing rate – one is able to carry on a conversation but does not have enough breath to sing (moderate), or is not able to carry on a conversation but not out of breath (vigorous).<br>Some of these activities include cycling, swimming, roller blading, hiking, basketball, etc.
	**Child Sleep Habits**
18.	Think of the last 7 days. Which of these activities does your child do <u>an hour</u> before bedtime on a WEEKDAY? (You can check more than one option)<br>☐ Communicate with others online (e.g., WhatsApp, Telegram)<br>☐ Consume caffeinated drinks (e.g., coffee, tea, soft drinks)<br>☐ Eat heavy supper or snacks (e.g., a full meal)<br>☐ Play games on a smartphone/other digital device (e.g., Animal Crossing)<br>☐ Use social media (e.g., Facebook, Twitter, Instagram, Snapchat)<br>☐ Watch television (e.g., viewing programs, accessing internet, playing games)<br>☐ Others: ____ (Please specify)
19.	Which of these activities does your child do <u>an hour</u> before bedtime on a WEEKEND? (You can check more than one option)<br>☐ Communicate with others online (e.g., WhatsApp, Telegram)

- ☐ Consume caffeinated drinks (e.g., coffee, tea, soft drinks)
- ☐ Eat heavy supper or snacks (e.g., a full meal)
- ☐ Play games on a smartphone/other digital device
- ☐ Use social media (e.g., Facebook, Twitter, Instagram, Snapchat)
- ☐ Watch television (e.g., viewing programs, accessing internet, playing games)
- ☐ Others: ____ (Please specify)

20. How much sleep does your child usually get per WEEKDAY night? (Think about a normal day in the last week)

**Duration of sleep is the difference between the time they go to bed to the time they get up*

____hr ____min (Please estimate)

21. How much* sleep does your child usually get per WEEKEND night? (Think about a normal day in the last week)

**Duration of sleep is the difference between the time they go to bed to the time they get up*

____hr ____min (Please estimate)

22. What sleep guidelines do you currently set for your child?

- ☐ Nine–eleven hours of sleep per night
- ☐ Regular sleep routine (same sleeping and waking time every day)
- ☐ Remove digital devices from the bedroom
- ☐ Limit the use of digital devices 30 minutes before sleep
- ☐ No heavy meals or caffeinated drinks 2–3 hours before sleep
- ☐ None - I do not set guidelines
- ☐ Others: ______ (Please specify)

Child Information

23. ☐ Male

	◻ Female
24.	Age<br>___ years ___months (As of 1 Jan 2020)
25.	Does your child wear spectacles or contact lenses?<br>◻ Yes<br>◻ No
26.	What kind of CCA is your child engaged in?<br>◻ Sport<br>◻ Non-sport (Uniformed group/Clubs and Societies)
	**Parent Information**
27.	Relationship to child<br>◻ Father<br>◻ Mother<br>◻ Guardian
28.	Do you wear spectacles, or contact lenses, or have had corrective surgery?<br>◻ Yes<br>◻ No
29.	Highest education attained<br>◻ No formal education<br>◻ Primary school<br>◻ Secondary school<br>◻ Post-secondary institution (College, Polytechnic)

- ◻ University – Bachelor degree
- ◻ University – Post-graduate degree (Masters and above)

30. Nationality
- ◻ Chinese
- ◻ Foreigner

31. What city do you live in?

____

Part 2 Survey of hyperactive behavior in children

Please answer the following questions by selecting the appropriate option based on your children's recent reality.

Content	***Not at all***	***Just a little***	***Pretty much***	***Very much***
Restless and overactive				
Excitable, impulsive				
Disturbs other children				
Fails to finish things-short attention span				
Constantly fidgeting				
Inattentive, easily distracted				
Demands must be met immediately – easily frustrated				
Cries often and easily				

Mood changes quickly and drastically				
Temper outbursts, explosive and unpredictable behavior				

End of Survey

Thank you

Supplementary-C: Reliability and validity tests of the online questionnaire package

Before distributing the online questionnaire package, a comprehensive process of translation and back-translation for the questionnaires was carried out. The initial translation into Chinese was undertaken by two Ph.D. students specializing in kinesiology, who possessed a deep understanding of Chinese culture and societal contexts. Subsequently, the questionnaires were back-translated by an English language teacher and a graduate student with expertise in the English language and a strong familiarity with the cultural nuances of China. The research team then consolidated and finalized the questionnaire package based on these translations.

To ensure the validity of the questionnaire, a specialist validity test was executed. This involved item analyses of the scale questions and initial reliability assessments using a limited-scale distribution of the questionnaire. Following this, a larger-scale distribution of the questionnaire was conducted to validate its efficacy. validation process encompassed various dimensions, including construct validity, structural validity, convergent validity, and discriminant validity. The overall validity of the questionnaire was evaluated by physical educators from both colleges and elementary schools.

In essence, the reliability of the scale questions was evaluated through the computation of the Cronbach's coefficient, a widely used measure of internal consistency. Conversely, the validity of the questionnaire package was examined through both exploratory factor analysis and confirmatory factor analysis. These analyses were performed using software tools such as SPSS 26.0 and AMOS 23.0, which are standard tools for statistical analysis and structural equation modeling.

B-1 Overall validity of the questionnaire

Four specialists in the field of physical education and health, hailing from universities, were enlisted for their expertise, along with two active physical education teachers and

two parents. Their collective role was to assess and evaluate the content, structure, and overall effectiveness of the questionnaire. The outcomes of this evaluation revealed unanimous agreement among the specialists thought that the questionnaire could effectively reflect the survey's content. Additionally, they deemed the design and structure of the questionnaire to be logically constructed and well-conceived, as evidenced by an average rating of 8.88 out of a possible 10.00. This high rating indicated a strong degree of specialist validity for the questionnaire.

Furthermore, a consensus was reached among all the experts regarding the necessity and importance of integrating questions pertaining to the COVID-19 pandemic. Consequently, an additional inquiry was introduced into the questionnaire, which inquired about alterations in the average duration of a child's daily engagement in physical activity, sleep, and digital media. Specifically, this change was evaluated in relation to the period preceding the occurrence of the COVID-19 pandemic. This supplementary question was deemed essential for providing a comprehensive understanding of the evolving behaviors influenced by the pandemic.

Table S1 The results of specialist validity evaluation

**Specia list**	**Role / Title**	**The degree of reflection on the survey content** (Completely able, More able, able, general, unable)	**Design Reasonableness** (Very reasonable, More reasonable，reasonable, average, unreasonable)	**Structural Reasonableness** (Very reasonable, More reasonable, reasonable, average, unreasonable)	**Total Rating Score**	**The necessity of virus outbreak-related issues** （Very necessary, more necessary, necessary, average, not necessary）
1	Professor	Completely able	Very reasonable	Very reasonable	9	Very necessary
2	Associate Professor	Completely able	Very reasonable	Very reasonable	10	Very necessary
3	Lecturer	Able	Reasonable	More reasonable	8	Necessary
4	Lecturer	Able	Reasonable	Reasonable	9	Necessary
5	First Class Teacher	Completely able	Very reasonable	Very reasonable	10	Very necessary
6	Teacher	More able	Reasonable	Reasonable	8	Necessary
7	Parent	Completely able	Very reasonable	Very reasonable	9	Very necessary
8	Parent	Able	Reasonable	Reasonable	8	Very necessary

B-2 Reliability and validity tests of scale questions

B-2-1 Item Analysis

A comprehensive item analysis was performed on the dataset consisting of 407 valid cases. During this analysis, adjustments were made to questions that exhibited a reversed trend, and the directional consistency of all questions was harmonized. Subsequently, the cumulative scores of the 12 items within the scale were computed and arranged in ascending order of their total scores. Following statistical principles, the top 27% of the scores were categorized as the "high" grouping, while the bottom 27% formed the "low" grouping. The corresponding critical values for these groups were determined as 27 and 41, respectively. The calculated value of Cronbach's α for this process was found to be 0.86, which signifies a good level of internal consistency. Distinct records were maintained for both the high and low subgroups, in accordance with the critical values associated with each dimension. Independent samples t-tests were then employed to evaluate the statistical significance of the differences in means between the predictors for the high and low subgroups. By applying predetermined criteria and judgment standards, it was established that items SL-Q and SL-M did not meet the prescribed criteria. Consequently, these items were excluded from the questionnaire during this phase of analysis.

Table S2 Item analysis results

Items	Extreme group comparison	Correlation between items and total score		Homogeneity test	Results
	Decisive value	Total Correlation	Corrected Item-Total Correlation (CITC)	Cronbach's α if Item Deleted	
MB1	-23.614	0.790**	0.726**	0.838	Keep
MB2	-32.351	0.841**	0.787**	0.832	Keep
MB3	-36.441	0.858**	0.811**	0.831	Keep
MB4	-29.376	0.827**	0.772**	0.834	Keep
MB5	-21.591	0.732**	0.648**	0.843	Keep
MB6	-24.667	0.751**	0.671**	0.842	Keep
MG1	-11.777	0.478**	0.371**	0.862	Keep
MG2	-10.112	0.522**	0.42**	0.859	Keep
MG3	-13.679	0.554**	0.462**	0.856	Keep
MG4	-13.273	0.543**	0.447**	0.857	Keep
SL-Q	-3.132	0.183**	0.078**	0.875	Delete
SL-M	-2.764	0.142**	0.071**	0.871	Delete

Judgment Criteria	≥3.00	≥0.4	≥0.4	≤ α

[a] MB stands for the benefits of digital media and MG stands for the disadvantages of digital media. MG1—communication (e.g., messaging, video call); MG2—entertainment (e.g., social media, playing video games); MG3—basic growth; MG4—participation in school-related activities (such as homework and project tasks); MB1—emotional health (e.g., unable to properly express and manage emotions); MB2—mental health (e.g., cyber bullying, access to inappropriate content); MB3—social health (e.g., lack of social interaction); MB4—physical health (e.g., lack of physical activity); MB5—child privacy (e.g., merchant data theft); MB6—safe (such as making friends with strangers, contact fraud); SL-M—the frequency child got up from sleep every night to watch electronic devices; SL-Q—child's sleep quality. ** represents $p \leq 0.01$

B-2-2 Reliability Analysis

The outcomes of the analysis indicated that the overall internal consistency reliability of the entire scale, denoted as α total, amounted to 0.887. This value surpasses the widely recognized threshold of 0.8, thereby affirming a commendable level of internal consistency for the scale's questions within the questionnaire package. Upon closer examination, the reliability assessment for each specific dimension revealed that α_{MB} (α for dimension MB) equated to 0.931, while α_{MG} (α for dimension MG) stood at 0.760. Both of these values exceed the minimum criterion of 0.6. This signifies that the questions encompassed in the scale exhibit a satisfactory level of reliability overall, enabling the subsequent progression of the validity analysis for the questionnaire.

Table S3 Reliability test results for each indicator of the scale

latent variable	Cronbach's α	Items Number
MB	0.760	6
MG	0.931	4
Total	0.887	10

[a] MB stands for the benefits of digital media and MG stands for the disadvantages of digital media.

B-2-3 Validity analysis

Following the initial positive outcomes, an additional 1000 questionnaires were gathered to undertake a more in-depth analysis of the scale's validity. To assess the validity of the scale questions, exploratory factor analysis was conducted using SPSS 23.0. This analysis aimed to validate the appropriateness of the dimensions within the scale. Subsequently, a confirmatory factor analysis was executed utilizing the AMOS 23.0 statistical software. This more advanced analysis sought to evaluate the construct validity, convergent validity, and discriminant validity of the scale.

The outcomes of exploratory factor analysis indicated that the value of the Kaiser-Meyer-Olkin (KMO) value stood at 0.874, exceeding the threshold of 0.8 with a significant p-value ($p<0.01$). Furthermore, the Cumulative Percentage of Variance achieved a value of 68.36%, surpassing the minimum requirement of 60%. These results demonstrate that the factors retained in the scale exhibited a substantial level of appropriateness and construct validity. The construct validity assessment, conducted through the confirmatory factor analysis, further validates the reliability and appropriateness of the dimensions within the scale. This thorough examination of the scale's validity enhances our confidence in its ability to accurately measure the intended constructs.

Table S4 Results of confirmatory factor analysis

Dimensions	Items	Communality	Factor loading	Results
MB	MB1	0.708	0.873	Reserved
	MB2	0.801	0.856	Reserved
	MB3	0.797	0.844	Reserved
	MB4	0.755	0.838	Reserved
	MB5	0.704	0.836	Reserved
	MB6	0.711	0.809	Reserved
MG	MG1	0.658	0.81	Reserved
	MG2	0.38	0.808	Reserved
	MG3	0.692	0.785	Reserved
	MG4	0.632	0.575	Reserved
	Cumulative % of Variance			68.396%

[a] MB stands for the benefits of digital media and MG stands for the disadvantages of digital media.

The results of the validated factor analysis revealed certain associations among dimensions that required further refinement. Initially, the $\chi 2$ /df ratio of the initial scale model exceeded the threshold of 5, suggesting the need for adjustments. Notably, emotional health was found to correlate with mental health within the dimension of "MB," while the dimension "children's privacy" exhibited an association with "safety." Similarly, the dimension "entertainment" displayed an association with "school activities" within the benefits of digital media use. These associations were logically sound and aligned with previous research. Given the reasonable justifications for these correlations, the model was subsequently revised to enhance its accuracy. Following the adjustments, the

corrected χ2 /df ratio improved to 3.25 (below the 5 threshold) and the root mean square error of approximation (RMSEA) reduced to 0.0475 (below the 0.08 cutoff), indicating a strong model fit. Although the root mean squared residual (RMR) was 0.05 (at the edge of 0.05 and below <0.1), other fit indices, such as the goodness of fit index (GFI), comparative fit index (CFI), normative fit index (NFI), tucker lewis index (TLI), and incremental fit index (IFI), were all greater than 0.9 (as depicted in Table 5—1–8). These results confirmed that the fit indices adhered to the criteria for good model fit, ultimately affirming the scale's structural validity.

Furthermore, the scales exhibited satisfactory convergent and composite validity, as evidenced by combined reliability (CR) values exceeding 0.7 and average variance extracted (AVE) values surpassing 0.36. Notably, the factor loadings of all questions in the scales were above 0.4, and the factor loadings between dimensions reached 0.46, all of which were statistically significant. Additionally, the factor loadings between dimensions remained below the square root of AVE, further establishing the scale's robust discriminant validity.

Table S5 Fitted indicators of the scale correction model

Fit Index	χ^2 /df	GFI	RMSEA	RMR	CFI	NFI	TLI	IFI
M	3.28	0.98	0.0475	0.05	0.989	0.984	0.984	0.989
Judgment Criteria	＜5	＞0.9	＜0.08	< 0.05	＞0.9	＞0.9	＞0.9	＞0.9

[a] GFI—Goodness of Fit Index；RMSEA—Root Mean Square Error of Approximation； RMR—Root Mean Squared Residual； CFI=—Comparative Fit Index； NFI—Normative Fit Index； TLI—Tucker–Lewis Index； IFI—Incremental Fit Index.

Table S6 Standard loadings, CR, and AVE values for each factor of the factors

latent variable	Observation variables	Standard load	AVE	CR
MB	MB1	0.81	0.67	0.92
	MB2	0.87		
	MB3	0.92		
	MB4	0.86		
	MB5	0.71		
	MB6	0.72		
MG	MG1	0.68	0.48	0.78
	MG2	0.56		
	MG3	0.77		

MG4	0.74	

[a] MB stands for the benefits of digital media and MG stands for the disadvantages of digital media.

Table S7 Results of discriminant validity

	F1	F2
F1	0.67	
F2	0.46	0.48
The square root of AVE	0.82	0.69

[a] MB stands for the benefits of digital media and MG stands for the disadvantages of digital media.

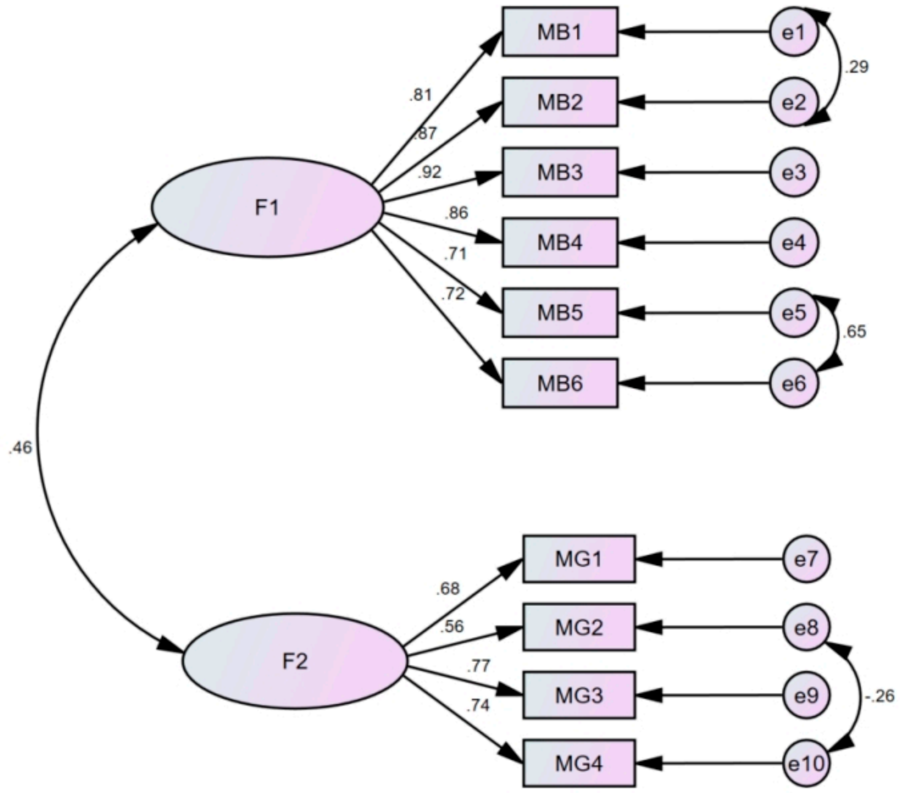

Figure S1. Fit diagram of the confirmatory factor model. F1 represents MB and stands for the benefits of digital media, F2 represents MG and stands for the disadvantages of digital media. MB1-6 and MG1-4 represent the specific items of each dimension
